# Fractionation and Purification of Bioactive Compounds Obtained from a Brewery Waste Stream

**DOI:** 10.1155/2013/408491

**Published:** 2013-05-12

**Authors:** Letricia Barbosa-Pereira, Ainara Pocheville, Inmaculada Angulo, Perfecto Paseiro-Losada, Jose M. Cruz

**Affiliations:** ^1^Department of Analytical Chemistry, Nutrition and Food Science, Faculty of Pharmacy, University of Santiago de Compostela, 15782 Santiago de Compostela, Spain; ^2^GAIKER Technological Centre, 48170 Zamudio, Spain; ^3^Department of Chemical Engineering, Industrial Engineering School, University of Vigo, 36310 Vigo, Spain

## Abstract

The brewery industry generates waste that could be used to yield a natural extract containing bioactive phenolic compounds. We compared two methods of purifying the crude extract—solid-phase extraction (SPE) and supercritical fluid extraction (SFE)—with the aim of improving the quality of the final extract for potential use as safe food additive, functional food ingredient, or nutraceutical. The predominant fractions yielded by SPE were the most active, and the fraction eluted with 30% (v/v) of methanol displayed the highest antioxidant activity (0.20 g L^−1^), similar to that of BHA. The most active fraction yielded by SFE (EC_50_ of 0.23 g L^−1^) was obtained under the following conditions: temperature 40°C, pressure 140 bar, extraction time 30 minutes, ethanol (6%) as a modifier, and modifier flow 0.2 mL min^−1^. Finally, we found that SFE is the most suitable procedure for purifying the crude extracts and improves the organoleptic characteristics of the product: the final extract was odourless, did not contain solvent residues, and was not strongly coloured. Therefore, natural extracts obtained from the residual stream and purified by SFE can be used as natural antioxidants with potential applications in the food, cosmetic, and pharmaceutical industries.

## 1. Introduction

Bioactive phenolic compounds are widely distributed in nature and are the most abundant antioxidants in the diet, being common components of fruits, vegetables, and beverages [1, 2]. Numerous studies have associated the consumption of foods rich in bioactive compounds, such as phenolic compounds, with the prevention of cardiovascular diseases, certain types of cancer, and other diseases related to aging [3]. The beneficial effects derived from phenolic compounds have been attributed to their antioxidant activity. These bioactive compounds may be a major determinant of the antioxidant potentials of foods, and they may therefore be a natural source of antioxidants [4]. Antioxidants are widely used in food products to prevent or delay the oxidation of fats and oils [5]. The recent worldwide trend to avoid or at least reduce the use of synthetic additives, such as BHT and BHA, has created the need to identify natural (and possibly safer) alternative sources of food antioxidants [6, 7]. In recent years, there has been a growing interest in the use of natural antioxidants in the food industry, not only for application as preservatives but also because of their benefits to human health [8, 9].

Beer production is an extensively studied biotechnological process that generates various by-products. The most common byproducts are generated from the main raw materials used to make beer, that is, barley malt, hop, and yeast. These by-products can be used in biotechnological processes, such as fermentative processes for the production of value-added compounds (e.g., xylitol, ethanol) as substrates for culturing microorganisms and as raw material for extraction of compounds such as antioxidants [10].

 Beer contains a large variety of phenolic compounds which are derived from the biotechnological fermentation of barley malt (70%) and hop (30%) and which are responsible for the overall antioxidant activity of the beverage [11, 12]. Numerous studies have shown that polyphenols are extremely important for the physical stability (a fundamental quality parameter) of beer [12]. During storage of beer, colloidal haze forms as a result of the complexes that polyphenols form with proteins and polypeptides [13]. The negative impact of malt and hop polyphenols on haze stability can be minimized by using polyvinylpolypyrrolidone (PVPP) resin to stabilize beer and consequently extend its shelf life. Stabilization with PVPP removes a substantial portion of the haze active and nonhaze active polyphenols from beer, and these polyphenols can subsequently be recovered from the PVPP by an alkaline treatment [14]. Therefore, a natural extract containing bioactive phenolic compounds with high antioxidant activity can be obtained from the alkaline residual stream generated after cleaning the PVPP in the brewery industry, by extraction in a solvent such as ethyl acetate [15].

The composition of the extract will depend on the solvent used and also on the quality of the original material, its composition, genetic factors, environmental conditions, storage conditions, and any prior treatment. In order to obtain a high quality extract with antioxidant activity that is suitable for use in the food, cosmetic, and pharmaceutical industries, the extract must be purified to remove all inert and undesirable components, so as to improve the antioxidant activity of the extract and minimize any odour, taste, and colour [16].

A purification process that removes fractions with limited antioxidant activity enables a good level of antioxidant activity to be obtained from relatively small amounts of the original natural extract. Moreover, it is also important to obtain pure extracts to ensure the identity and safety of antioxidant compounds to be used as food additives [17].

In the present study, we evaluated two methods of purifying the crude extract—solid-phase extraction (SPE) and supercritical fluid extraction (SFE). SPE has been widely used for clean-up and purification of extracts as well as preconcentration of juices, wines, and beer. Phenolic compounds are readily fractionated by several formats of SPE in different materials of natural origin; elution with methanol on reverse-phase columns is the most popular method of separating these compounds [18–20].

Extraction and recovery of valuable compounds are the most common uses for SFE, which operate at low temperatures, in the absence of oxygen, and typically use CO_2_ as extraction solvent (SC-CO_2_). These features make SFE an ideal technique for extracting bioactive compounds [21]. The most obvious advantages of SFE are that it is clean and environmentally friendly. Direct SC-CO_2_ extraction is not recommended for by-products obtained on a large scale and that contain small amounts of bioactive compounds [17]. However, SFE has been used to purify crude extracts yielded by organic solvents, to improve their purity and their biological properties without thermal or chemical degradation. As CO_2_ is a non-toxic, inexpensive, noninflammable, volatile solvent, it can be used in a variety of different conditions [22, 23]. The extraction efficiency of SC-CO_2_ can be optimized by changing the density of CO_2_ (varying pressure and temperature), the modifier (e.g., organic solvent), modifier percentage, or time, among other parameters. Due to the apolar nature of CO_2_, the use of modifiers (e.g., ethanol) can significantly improve the recovery of the phenolic compounds due to the polarity of these compounds [17].

The aims of the present study were (i) to evaluate the efficiency of the SPE and SFE techniques to purify natural antioxidants obtained from brewery waste and (ii) to determine the recovery yield and the radical-scavenging activity of the fractions obtained. Chemical analysis of the fractions by reversed-phase high-performance liquid chromatography (RP-HPLC) coupled to a diode array detector (DAD) was carried out to identify and quantify the polyphenols responsible for the antioxidant activity.

## 2. Materials and Methods

### 2.1. Reagents, Solvents, and Standard Phenolics

Ethyl acetate (GR for analysis), methanol (≥ 99.9%), absolute ethanol, hydrochloric acid (37%), glacial acetic acid, and acetonitrile (ACN, HPLC grade) were obtained from Merck (Darmstadt, Germany). Ultrapure water was prepared using a Milli-Q filter system (Millipore, Bedford, MA, USA). 2,2-diphenyl-1-picrylhydrazyl (DPPH, ≥ 85%) and gallic acid (≥ 98%) were supplied by Fluka Chemie AG (Buchs, Switzerland). 2,6-Di-tert-buthyl-4-methylphenol (BHT, 99.0%) and 2(3)-tert-butyl-4-hydroxyanisole (BHA, 98%) were provided by Sigma-Aldrich (Steinhein, Germany). Supercritical carbon dioxide, CO_2_ SCF (purity : 99.998%), was supplied by Air Liquide (Spain).

Polyphenol standards were supplied as follows: protocatechuic acid (≥ 97.0%), caffeic acid (≥ 98.0%), (−)-epicatechin (≥ 90%), acetosyringone (97%), resveratrol (≥ 99%), (±)-naringenin (95%), epigallocatechin (≥90%), (+)-catechin hydrate (98%), ferulic acid (99%), quercetin (≥ 98%), kaempferol (≥97.0%), gallocatechin (≥ 98%), p-coumaric acid (≥ 98.0%), and apigenin (≥ 97%) by Sigma-Aldrich (Steinhein, Germany); gallic acid (≥ 98.0%), syringic acid (≥ 97%), isoquercetin, and salicylic acid (≥ 99.0%) by Fluka Chemie AG (Buchs, Switzerland); and homovanillic acid (98%), 4-hydroxybenzoic acid (99%), and acetovanillone (98%) by Alfa Aesar (Karlsruhe, Germany).

### 2.2. Sampling

**Figure 1:**
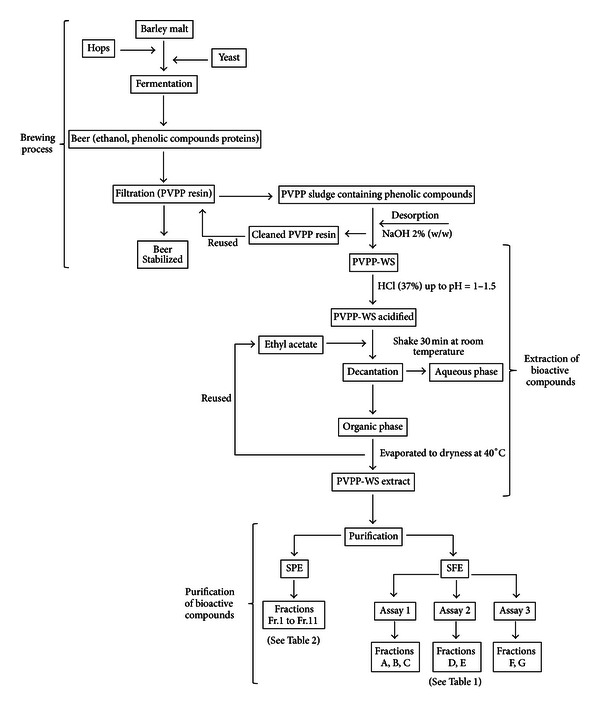
Schematic representation of the brewing process, extraction, and purification of the PVPP-WS extract containing bioactive compounds.

In beer production, a clarification step is essential to improve beer stability. As a result of this process, a PVPP sludge is obtained in the brewing industry. The PVPP sludge loaded with polyphenolic compounds was washed with a NaOH solution (2% w/w) at room temperature. After the NaOH-PVPP was filtered, a cleaned PVPP resin and a PVPP washing solution (PVPP-WS) containing phenolic compounds were obtained (see Figure 1). The residual stream generated after the PVPP cleaning process was kindly supplied by Mahou-San Miguel, Spain.

### 2.3. Industrial Plant Scale Extraction of the Antioxidants from PVPP Sludge

The PVPP-WS (1000 L) was acidified to pH 1.5 with HCl (37%), and polyphenolic compounds were extracted with ethyl acetate (2000 L) by stirring for 30 minutes at room temperature. The organic and aqueous phases were separated by decantation, and the organic phase was collected and evaporated to dryness at 40°C. The residual water was removed from the extract by lyophilisation before the recovery yield was determined gravimetrically, and the dry extract was used in fractionation experiments (see Figure 1).

### 2.4. Fractionation and Purification of PVPP Crude Extract

#### 2.4.1. Solid-Phase Extraction (SPE)

SPE was performed with super clean cartridges (LC-18 20 mL, from Supelco, Germany) and 5 g of reversed-phase sorbent (modified silica with octadecyl groups). The crude extract (50 mg) was dissolved in 10 mL of water and loaded on the cartridge. The natural extract was eluted with different percentages of methanol (v/v): 0%, 10%, 20%, 30%, 40%, 50%, 60%, 70%, 80%, 90%, and finally 100% of methanol, so that eleven separate fractions were obtained at the end of the process. All fractions were evaporated to dryness, under vacuum at 40°C, in a rotary evaporator, and finally redissolved in methanol for further analysis. The recovery yield of each fraction was determined gravimetrically, and the antioxidant activity of each fraction was measured by the DPPH radical-scavenging test. The phenolic compounds responsible for the antioxidant activity were determined by HPLC-DAD.

#### 2.4.2. Supercritical Fluid Extraction

The crude extract was fractionated using a supercritical fluid SCF R100 system (Thar Technologies, Inc.) equipped with a 5 mL SFE cell (Thar Technologies, Inc.).

Different extraction conditions were tested in three different assays. In each assay, 1 g of sample was submitted to the fractionation procedure. The assay conditions are shown in Table 1.

*Assay 1—Pressure Mode (Pressure Range).* The experiment was run at 40°C, and CO_2_ was automatically fed into the system by the CO_2_ pump to maintain a constant pressure. For fractionated separation of the different compounds present in the crude extract, pressure of between 100 and 300 bar (100, 120, 140, 160, 200, 250, and 300 bar) was applied. The extraction time was 30 minutes at each pressure tested. Experiments were carried out with and without modifier. Ethanol was used as a modifier, and two different flow rates (0.1 mL min^−1^ and 0.2 mL min^−1^) were tested.

*Assay 2—Flow Mode (Pressure Range).* The trial was run at 40°C, and CO_2_ was fed into the system at a flow rate of 3 g min^−1^. The pressure range and extraction time at each pressure tested were the same as described earlier. The modifier (ethanol) was applied under different conditions: 3% and 6% of modifier at flow rate of 0.1 mL min^−1^ and 0.2 mL min^−1^, respectively.

*Assay 3—Flow Mode (Percentage of Modifier Range).* The experiment was run at 40°C, and CO_2_ was fed into the system at a flow rate of 3 g min^−1^. In this test, pressure was maintained constant at 140 bar. Two modifiers, ethanol and methanol, were tested within a range of 0 and 3% (0%, 0.5%, 1.0%, 1.5%, 2.0%, 2.5%, 3.0%). 

Single extracts obtained under the three different test conditions were evaporated to dryness under nitrogen steam. The recovery yield of each fraction was determined gravimetrically. Fractions were characterized by HPLC-DAD, and the antioxidant activity of each was determined by the free radical method DPPH.

### 2.5. Separation and Quantification of Bioactive Phenolic Compounds (RP-HPLC-DAD)

Chromatographic analysis was performed on an HPLC system model 1200 HP (Hewlett-Packard, Waldbronn, Germany), equipped with a diode array detector (DAD) and controlled by HP Chemstation chromatographic software.

Chromatographic separation of polyphenols was carried out on a reverse phase Kromasil C18 column (250 × 3.2 mm internal diameter, 5 *μ*m particle size) (Phenomenex, Barcelona, Spain). The solvents constituting the mobile phase were milli-Q water 0.1% acetic acid (solvent A) and 100% ACN (solvent B). The gradient program was as follows: 0–5 min  , 90% A and 10% B; 5–35 min, linear gradient until reaching 50% B at 35 min; 35–43 min, 50% B isocratic; 43–45 linear gradient from 50% to 10% B; and finally, the column was washed and reconditioned. The mobile phase flow rate was 0.5 mL min^−1^ during the entire analytical run, the column temperature was set at 38°C, and the sample injection volume was 20 *μ*L. A scan in the range of 190 to 700 nm was continuously performed, by DAD.

Individual phenolic compounds were identified by comparing their retention time and their UV spectrum with those obtained by injecting standards in the same HPLC conditions. Phenolic acids were monitored and quantified at 225 nm, flavan-3-ols, flavanones, flavones, and acetophenone derivates at 280 nm, hydroxycinnamic acids and resveratrol at 325 nm, and flavonols at 372 nm.

### 2.6. Antioxidant Activity, DPPH Assay

The antioxidant activity of phenolics in the crude extract and its fractions, obtained during the purification processes, were determined by the DPPH (2,2-diphenyl-1-picrylhydrazyl) radical scavenging method described by von Gadow et al. (1997) with slight modifications [24].

Standard solutions of the different antioxidant fractions and of two synthetic compounds with antioxidant properties, BHA and BHT, which are commonly used in the food industry, were prepared in methanol. An aliquot of antioxidant (50 *μ*L) was added to 2 mL of DPPH radical methanolic solution (3.6 × 10^−5^ M), shaken vigorously on a vortex shaker (MS2 Mini Vortex Shaker IKA), and left to stand in the dark at room temperature. The absorbance was measured at 515 nm, after 16 min at room temperature, in a dual-beam spectrophotometer (Uvikon XL, Bio-Tek Instruments, Milan, Italy). All determinations were performed in triplicate. The decrease in absorbance was converted to inhibition percentage of the DPPH (IP), according to the following equation:

(1) $$\begin{array}{c} \text{IP}=\frac{A_{0}-A_{16}}{A_{0}}\times 100, \end{array}$$

where *A*_0_ is the absorbance of the control at initial time; *A*_16_ is the absorbance of the sample after 16 minutes.

The concentration of antioxidant compound or fraction required to achieve 50% inhibition of the radical DPPH (equivalent concentration = EC_50_) was determined from the linear regression curve obtained by plotting the different concentrations of antioxidant compound or fraction used (within the range 0.1 to 3.5 g L^−1^) against the inhibition percentage of the DPPH (IP). 

## 3. Results and Discussion

In this study, a new by-product (PVPP-WS) was considered as a natural source of antioxidant-rich bioactive compounds with several potential applications.

The extraction procedure used to obtain the crude extract has already been tested at laboratory scale and pilot plant scale in previous studies. With the overall aim of enabling the brewing industry to implement this extraction process in industrial plants, the present study investigated the scaling-up of the extraction process and the purification of the bioactive phenolic compound extracted. The extraction yield was approximately 0.1%. In the brewery industry, around one litre of this waste stream (PVPP-WS) can be generated from every 138 L of beer produced. Approximately 403 million hectolitres of beer were produced in Europe in 2010, which means that up to 400 tons of this crude extract could be obtained in Europe every year [25].

The crude extract must be processed (by purification and fractionation) as its brown colour would hinder its use as a food additive. In addition, more information about the composition of the crude extract in bioactive phenolic compounds could be obtained from different fractions to determine the correlation between the antioxidant activity and the phenolic compounds or group of phenolic compounds present in the fractions.

### 3.1. Solid-Phase Extraction Results

Solid-phase extraction is generally used for sample clean-up, fractionation, purification and/or preconcentration of natural extracts. In this study, eleven differently coloured fractions containing phenolic compounds were obtained (see Figure 1).

#### 3.1.1. Extraction Yield and Antioxidant Activity

The recovery yield and the radical scavenging activity were determined for each fraction obtained at each solvent ratio applied to the cartridge. The results are shown in Table 2, along with the colour of each fraction. The antioxidant activity of the synthetic antioxidants commonly used in the food industry was also evaluated to compare the potential of the natural compounds as food additives. The crude extract was also tested to evaluate whether the fractionation process yielded fractions that were more or less active than the crude extract.

The most active fractions and the best yields obtained in the SPE process corresponded to the first seven fractions eluted with a solvent mixture from 0%–60% of methanol. This showed that the natural extract and the polyphenolic compounds are water soluble but the addition of methanol yielded the most active fractions. Therefore, the fraction obtained with 30% (v/v) of methanol exhibited the highest antioxidant activity. All the fractions that display a notable level of antioxidant activity (fractions 1–7) were coloured, particularly fractions Fr. 4, Fr. 5, and Fr. 6, which also displayed the highest degree of antioxidant activity against the free radical DPPH. This is consistent with the results described by Woffendem et al. in a study evaluating the relationship between antioxidant activity and colour of crystal malt extracts [26]. Fractions 8, 9, 10, and 11 were colourless, and the antioxidant activity was lower than that of the other extracts.

The EC_50_ values of Fr. 3, Fr. 4, Fr. 5, and Fr. 6 were similar to that of the synthetic antioxidant BHA used in food industry. Except for the last 4 fractions yielded, all the fractions obtained from the crude extract showed a higher DPPH radical scavenging capacity than the antioxidant BHT, also commonly used in food industry. In this study, the antioxidant capacity of the crude extract (EC_50_ = 0.32 g L^−1^) was also calculated: (a) to compare the capacity of this extract and the fractions obtained; and (b) to evaluate whether the antioxidant activity increased as a result of the purification process. Purification of the crude extract yielded fractions with higher antioxidant activity than the crude extract.

#### 3.1.2. HPLC-DAD-UV Analysis of the Antioxidant Fractions

The results of the identification and quantification of major polyphenolic compounds present in each fraction obtained in the fractionation process (SPE) by HPLC-DAD-UV are shown in Table 3.

The different ratios of solvents yielded different fractions. These fractions displayed different levels of antioxidant activity because the polyphenolic content varies considerably with solubility.

The first fractions, which exhibited the highest level of antioxidant activity (see Table 2), contained the highest amounts of polyphenolic compounds (Table 3). Fractions 1 and 2 contain large amounts of gallocatechin, which largely accounts for the high radical scavenging activity exhibited by these two fractions. Compounds with flavonoid structure such as catechin generally display a higher level of antioxidant activity than nonflavonoid compounds [27].

Fraction 4 displayed the highest level of antioxidant activity, mainly due to the high content of ferulic acid, a phenolic compound. However, Fraction 3, which displayed a similar level of antioxidant activity to Fraction 4, also contains large amounts of antioxidant compounds such as epigallocatechin, caffeic acid, p-coumaric acid, and isoquercetin [11]. Hydroxycinnamic and hydroxybenzoic acids are known antioxidants that act as free radical acceptors and chain breakers [28]. Caffeic, ferulic, and p-coumaric acids have been widely studied and reported to be major contributors to the antioxidant activity of beer [27, 29]. In the present study, the natural extract was obtained after the treatment of the beer, and, as expected, this crude extract was loaded with these bioactive compounds. Fractions 2 and 3, which contain these compounds, were the most active free radical scavengers. These findings may be attributed to a combined, synergistic, and/or additive action. This type of action has previously been observed to result in an increased antioxidant potential [30].

The recovery yields and the phenolic contents of fractions 6 and 7 were lower than those of the other fractions. However, both of these fractions displayed some antioxidant activity, mainly due to the flavonols. Fractions 6 and 7 contain the flavonols quercetin and kaempferol at similar concentrations. Fractions 8, 9, 10, and 11 were extracted using high contents of methanol, and no phenolic compounds were detected in the fractions. 

### 3.2. Supercritical Fluid Extraction

SC-CO_2_ has been used successfully to purify crude extracts by concentrating the bioactive compounds (e.g., antioxidants) in the extract and also by removing contaminants. In the present study, several fractions were obtained from the SFE fractionation assay under different test conditions (see Figure 1).

#### 3.2.1. SFE Fractions (Extraction Yield, Colour, and Antioxidant Activity of Each Fraction)

The extraction yield of the different antioxidant fractions obtained under the different operational conditions (see Table 1) and the colour of each fraction are shown in Table 4. The extraction yield ranged from 0.12% (fractionation test A), for the extraction without cosolvent (see Table 4(a)), to 41.5% (fractionation test E) for the extractions in which ethanol was used as a modifier (see Table 4(b)). The results showed that the use of a modifier is mandatory essential for successful fractionation of the crude extract. This is consistent with data reported by various authors who obtained high extraction yields from diverse natural matrices by using modifiers [17].

Results showed that the increase in the percentage of modifier increases the amount of extract (see Table 4(b)). The volume of the extract collected at each pressure is more constant in the flow mode (see Table 4(b), tests D and E) than in pressure mode (see Table 4(a), tests B and C). Moreover, when higher amounts of the modifier are used, less pressure is required, as observed in tests C (flow rate of 0.2 mL min^−1^ twice that of B) and E (6% of modifier twice that of D), in which almost all compounds were yielded at a pressure of 120 bar. These results indicated that the optimal conditions for the extraction process of the crude extract are those used in test E, which yielded most phenolic compounds and fractions with the highest antioxidant activities (see Table 5) similar to BHA and higher than that of BHT (see Table 2). Moreover, under these conditions, the fractions were paler, which is an advantage as the strong brown colour of the crude extract may hinder its use as a food additive. However, the fractions obtained in high amounts were always darker, as, for example, E_120_ (see Table 4(b)).

The darkest fractions, which contained more phenolic compounds per weight of extract fraction (see Tables 4(a), 4(b) and 4(c)), displayed a high radical scavenging capacity (see Table 5). Colourless fractions, which did not appear to contain phenolic compounds, did not display any scavenging activity of the radical DPPH. This is further indication that fractions colour depends on the content of active compounds, which are also responsible for the antioxidant activity of the fractions.

In the present study, methanol was also evaluated as a modifier in test G (see Table 4(b)). The results showed that ethanol is more suitable for the extraction process because the phenolic compounds of interest were obtained with a lower percentage of ethanol (1.5%), while 2% methanol was required to obtain compounds such as ferulic acid and gallocatechin (data not shown), the main compounds present in the crude extract. Selection of a suitable modifier and/or adjustment of the modifier percentage is essential for successful fractional separation of the antioxidant compounds present in the crude extract.

#### 3.2.2. HPLC-DAD-UV Analyses of the Antioxidant Fractions

The fractions obtained by SFE were analysed by HPLC-DAD-UV to the characterization of bioactive compounds in the crude extract.

**Figure 2:**
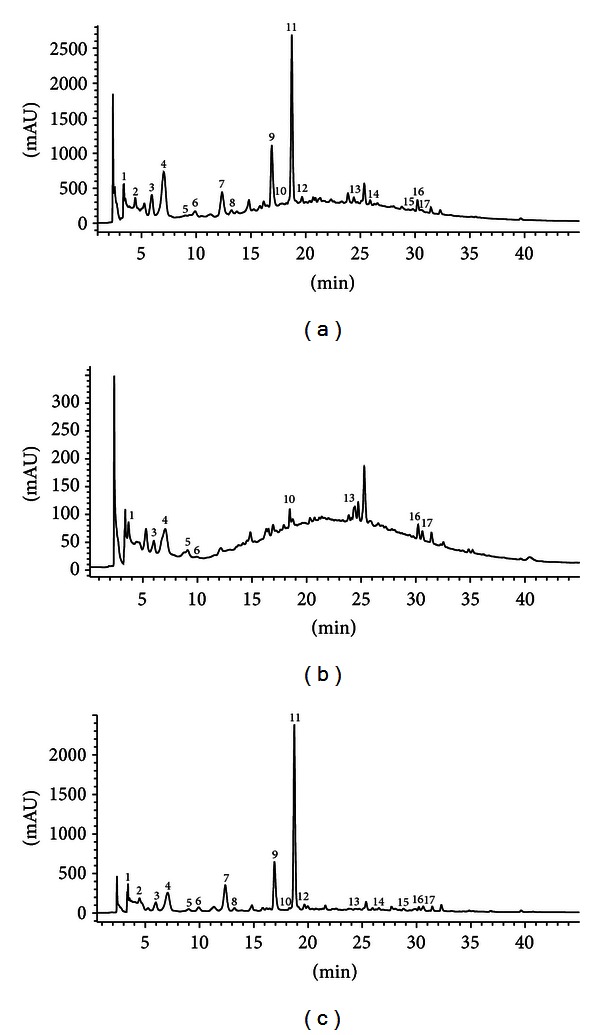
Chromatograms acquired at 280 nm  by HPLC-DAD-UV. (a) Chromatogram of the crude extract without fractionation process. (b) Chromatogram of the residue of the crude extract that remains in the extraction cell after the fractionation process (E_Cell_). (c) Chromatogram of the fraction obtained under optimal conditions (E_140_).

Figure 2 shows, as a representative example, the chromatogram of the crude extract before fractionation by SFE (Figure 2(a)) and the chromatograms obtained after the fractionation procedure under optimal conditions, the extraction cell (Figure 2(b)) and the fraction E_140_ (Figure 2(c)). Chromatogram C (Figure 2(c)) shows that SFE fractionation improves the purity of the phenolic compounds (clearly observed from the base line of the chromatogram) relative to the crude extract (Figure 2(a)). It is also evident that the impurities in the crude extract remain in the extraction cell, as verified by the peak obtained in chromatogram B. However, phenolic compounds and probably other compounds not identified in the present study remain active in the extraction cell (E_Cell_), which confers antioxidant activity to the residue in the extraction cell (see Table 5).

The phenolic compounds present in the fractions obtained under optimal conditions in this study (fractionation E) are shown in Table 6. The number of phenolic compounds shown in the table corresponds to the numbers in the chromatograms in Figures (2(a), 2(b), and 2(c)). The phenolic compounds were identified and quantified in this study for all the fractions obtained under the different assay conditions (data not shown). The qualitative composition of phenolic compounds in the factions yielded by SFE is the same in almost every fraction obtained under the different test conditions assayed (see Table 6). The main bioactive phenolic compounds identified in the fractions were as follows: ferulic acid, p-coumaric acid, caffeic acid, protocatechuic acid, catechin, gallic acid, gallocatechina, and epigallocatechin. These results are consistent with those obtained with the SPE procedure (see Section 3.1.2.). 

The phenolic compounds were not successfully separated by the SFE fractionation procedure as in the SPE fractionation (LC-18 column). However, the purity of the fractions yielded by the SFE was greater than that of the fractions yielded by SPE. Moreover, large amounts of pure fractions containing the main bioactive phenolic compounds were obtained by SFE, which makes this technique the most promising for purification of the crude extract.

Regarding the antioxidant activity, the fractions with the highest contents of polyphenolic compounds are those with the highest antioxidant activity. However, the antioxidant activities of the different fractions did not differ significantly because the composition of bioactive compounds was also very similar.

## 4. Conclusions

Industrial extraction of a natural extract containing bioactive compounds was successful and could be implemented in the brewing industry to recover a residue with added value. The dried ethyl acetate crude extract was purified by two alternative procedures to improve its quality (antioxidant activity and organoleptic properties) for potential use as a food additive.

Both purification procedures yielded fractions with better organoleptic properties (odour and colour) and with higher antioxidant activity than the crude extract. The fractions that display strong antioxidant activity may be suitable for use as food additives (to increase the shelf life of food by preventing lipid peroxidation and protecting from oxidative spoilage during storage). Moreover, these bioactive compounds may be a good source of compounds with several applications in the food industry, as food ingredients and nutraceuticals, in the cosmetics, and in pharmaceutical industries.

## Figures and Tables

**Table 1:** SFE operational conditions tested.

Assay	*T* (°C)	Time (min)	CO_2_ flow (g min^−1^)	Pressure (bar)	Modifier	Modifier (%)	Modifier flow (mL min^−1^)	Fractions obtained
					None	None	None	A_100_, A_120_, A_140_, A_160_, A_200_, A_250_, A_300_
1	40	30	—*	100, 120, 140, 160, 200, 250, 300	Ethanol	—*	0.1	B_100_, B_120_, B_140_, B_160_, B_200_, B_250_, B_300_
						—*	0.2	C_100_, C_120_, C_140_, C_160_, C_200_, C_250_, C_300_

2	40	30	3	100, 120, 140, 160, 200, 250, 300	Ethanol	3 6	0.1 0.2	D_100_, D_120_, D_140_, D_160_, D_200_, D_250_, D_300_ E_100_, E_120_, E_140_, E_160_, E_200_, E_250_, E_300_

3	40	30	3	140	Ethanol Methanol	0, 0.5, 1, 1.5, 2, 2.5, 3	0.0, 0.02, 0.03, 0.05, 0.06, 0.08, 0.1	F_0_, F_0.5_, F_1_, F_1.5_, F_2_, F_2.5_, F_3_ G_0_, G_0.5_, G_1_, G_1.5_, G_2_, G_2.5_, G_3_

*In pressure mode, neither CO_2_ flow nor modifier percentage were controlled.

**Table 2:** Recovery yield, radical scavenging activity (DPPH), and colouration of each fraction (Fr.) obtained by SPE with different % of methanol.

Sample	% of methanol	Recovery yield (% w/w)^a^	DPPH (EC_50_)^b^	Colour
Fr. 1	0	10.4	0.44	Wheat
Fr. 2	10	18.1	0.30	Burnt orange
Fr. 3	20	16.2	0.27	Brown
Fr. 4	30	19.4	0.20	Maroon
Fr. 5	40	18.3	0.26	Dark brown
Fr. 6	50	8.19	0.23	Dark brown
Fr. 7	60	6.71	0.89	Ochre
Fr. 8	70	1.59	7.02	Colourless
Fr. 9	80	0.324	6.64	Colourless
Fr. 10	90	0.615	8.25	Colourless
Fr. 11	100	0.194	14.3	Colourless
Crude extract	—	—	0.32	Dark brown
BHA	—	—	0.24	—
BHT	—	—	2.67	—

^a^Values expressed as % of dry crude extract.

^b^Values expressed as g L^−1^ of extract (fraction).

**Table 3:** Phenolic compounds present in the different fractions, obtained with a LC-18 column, identified and quantified by HPLC-DAD.

	Fraction											Crude extract
Antioxidant compound	1	2	3	4	5	6	7	8	9	10	11	—
	mg g^−1^ of fraction											mg g^−1^
Gallic acid	193	—	—	—	—	—	—	—	—	—	—	20.1
Gallocatechin	178	641	—	—	—	—	—	—	—	—	—	132
Protocatechuic acid	—	72.9	9.51	—	—	—	—	—	—	—	—	15.5
Epigallocatechin		86.2	96.4									30.6
Catechin	—	114	75.5	—	—	—	—	—	—	—	—	29.9
4-Hydroxybenzoic acid	—	6.51	7.49	—	—	—	—	—	—	—	—	2.54
Caffeic acid	—	13.7	66.0	—	—	—	—	—	—	—	—	14.1
Epicatechin	—	—	132	—	—	—	—	—	—	—	—	21.4
p-coumaric acid	—	—	73.9	1.83	—	—	—	—	—	—	—	11.4
Isoquercetin	—	—	166	—	—	—	—	—	—	—	—	28.3
Ferulic acid	—	—	29.6	138	—	—	—	—	—	—	—	33.7
Acetosyringone	—	—	35.2	50.9	—	—	—	—	—	—	—	14.6
Resveratrol	—	—	—	—	—	51.6	14.5	—	—	—	—	5.35
Quercetin	—	—	—	—	31.7	117	—	—	—	—	—	14.5
Apigenin	—	—	—	—	—	58.8	59.7	—	—	—	—	8.10
Kaempferol	—	—	—	—	—	25.3	23.4	120	—	—	—	6.06
Naringenin	—	—	—	—	—	38.7	171	11.8	—	—	—	14.6

Total (mg g^−1^)	371	934	692	191	31.7	292	269	132	—	—	—	403

**Table:** (a)

*P* (bar)	A		B		C	
	Yield (%)	Colour	Yield (%)	Colour	Yield (%)	Colour
**100**	0.02	Colourless	0.24	Wheat	0.02	Colourless
**120**	0.02	Colourless	7.71	Dark brown	23.04	Dark brown
**140**	0.02	Colourless	4.27	Burnt orange	6.39	Brown
**160**	0.00	Colourless	2.77	Burnt orange	0.06	Wheat
**200**	0.02	Colourless	12.7	Maroon	0.01	Colourless
**250**	0.02	Colourless	8.09	Dark brown	0.07	Ochre
**300**	0.02	Colourless	3.29	Maroon	0.03	Colourless

Total	0.12%		39.1%		29.6%	

**Table:** (b)

*P* (bar)	D		E	
	Yield (%)	Colour	Yield (%)	Colour
**100**	0.89	Brown	3.27	Burnt orange
**120**	0.73	Brown	19.83	Dark brown
**140**	0.76	Brown orange	5.67	Burnt orange
**160**	1.42	Burnt orange	3.65	Burnt orange
**200**	1.13	Burnt orange	3.84	Burnt orange
**250**	0.79	Burnt orange	2.56	Burnt orange
**300**	0.98	Burnt orange	2.64	Burnt orange

Total	6.7%		41.5%	

**Table:** (c)

% Modifier	F		G	
	Yield (%)	Colour	Yield (%)	Colour
**0**	0.04	Orange	0.13	Wheat
**0.5**	0.07	Coral	0.08	Wheat
**1.0**	0.33	Wheat	0.31	Wheat
**1.5**	0.58	Wheat	0.28	Wheat
**2**	0.44	Wheat	0.58	Wheat
**2.5**	0.61	Wheat	0.41	Wheat
**3**	1.08	Wheat	0.59	Wheat

Total	3.15%		2.38%	

**Table 5:** Antioxidant activity of SFE extracts determined by DPPH. Results are expressed as EC_50_ (g L^−1^).

	Fraction								
*P* (bar)	Pressure mode			*P* (bar)	Flow mode		Flow mode		
	A	B	C		D	E	% modification	F	G
**Cell**	—	—	0.23	**Cell**	0.20	0.22	**Cell**	0.26	0.20
**100**	n.d.	2.65	37.73	**100**	0.33	0.74	**0**	21.99	0.64
**120**	n.d.	0.36	0.41	**120**	0.51	0.36	**0.5**	1.57	0.68
**140**	n.d.	0.27	0.25	**140**	0.62	0.23	**1.0**	0.59	3.52
**160**	n.d.	0.29	0.28	**160**	0.46	0.21	**1.5**	0.97	1.42
**200**	n.d.	0.34	1.00	**200**	0.74	0.22	**2**	0.88	0.80
**250**	n.d.	0.21	0.20	**250**	0.34	0.23	**2.5**	0.64	0.67
**300**	n.d.	0.10	3.12	**300**	0.42	0.34	**3**	0.54	0.42

**Table 6:** Phenolic profile of each fraction obtained under SFE conditions of test E: temperature 40°C, extraction time 30 minutes, modifier ethanol (6%), and modifier flow 0.2 mL min^−1^. Phenolic compounds are expressed as mg g^−1^ of fraction.

Peak no.	Phenolic compound	SFE fractions (mg g^−1^)							
		E_Cell_	E_100_	E_120_	E_140_	E_160_	E_200_	E_250_	E_300_
1	Gallic acid	1.04	0.24	1.05	1.68	2.61	3.02	3.44	3.07
2	Gallocatechin	171	10.7	14.8	33.9	41.0	33.3	29.8	20.9
3	Protocatechuic acid	—	1.99	4.12	9.19	12.8	14.9	16.7	16.0
4	Epigallocatechin	1.43	10.7	10.2	—	—	—	—	—
5	Catechin	6.10	3.40	3.36	6.88	11.8	11.7	15.7	16.7
6	4-Hydroxybeinzoic acid	—	3.58	1.96	4.78	4.22	2.72	2.36	1.55
7	Caffeic acid	—	7.29	7.38	17.1	19.65	14.8	13.3	9.52
8	Epicatechin	—	—	—	—	0.23	0.14	0.17	0.40
9	p-coumaric acid	—	7.97	5.89	13.5	13.9	9.66	8.45	6.26
10	Isoquercetin	2.51	—	—	—	—	3.71	3.75	1.43
11	Ferulic acid	—	35.7	27.6	69.0	70.1	47.0	38.9	22.0
12	Acetosyringone	—	2.92	0.83	3.62	2.07	1.18	3.10	2.55
13	Resveratrol	—	—	0.54	1.68	0.37	0.46	0.60	0.80
14	Quercetin	0.57	0.65	0.98	1.91	2.61	2.83	3.13	2.96
15	Apigenin	—	—	0.04	0.66	0.78	0.72	0.79	0.93
16	Kaempferol	1.48	0.94	1.65	3.17	4.42	3.18	6.24	6.64
17	Naringenin	0.39	0.40	0.78	1.69	2.45	2.75	3.26	3.52

	Total (mg g^−1^)	11.0	88.9	82.5	172.7	192.8	155.9	152.3	117.0
